# The Association between Cultural Orientation and Drinking Behaviors among University Students in Wuhan, China

**DOI:** 10.1371/journal.pone.0054796

**Published:** 2013-01-24

**Authors:** Hongxiu Tang, Weibin Cai, Hongjing Wang, Qing Zhang, Ling Qian, Duane F. Shell, Ian M. Newman, Ping Yin

**Affiliations:** 1 Department of Epidemiology and Biostatistics, School of Public Health, Tongji Medical College, Huazhong University of Science and Technology, Wuhan, China; 2 Chinese Health Education Center, Beijing, China; 3 Nebraska Prevention Center for Alcohol and Drug Abuse, University of Nebraska-Lincoln, Lincoln, Nebraska, United States of America; 4 Department of Educational Psychology, University of Nebraska-Lincoln, Lincoln, Nebraska, United States of America; Federal University of Rio de Janeiro, Brazil

## Abstract

**Objectives:**

This study examines the association between cultural orientation and drinking behaviors among university students. Cultural orientation is the measure of how the cultural values of individuals living in their own society are influenced by cultural values introduced from the outside.

**Methods:**

In 2011, a cross-sectional survey collected data from 1279 university students from six universities in central China. Participants used a likert scale to rank a series of statements reflecting cultural values from the previously validated Chinese Cultural Orientation Scale and answered questions about their drinking behaviors and socio-demographic characteristics.

**Results:**

Statistically significant differences in cultural orientation were observed for gender, hometown and type of university attendance. Traditional-oriented students were more likely to be occasional drinkers or nondrinkers, while marginal-oriented students, bicultural-oriented students and western-oriented students were more likely to be regular drinkers. Bicultural orientation (*OR* = 1.80, *P*<0.05) and marginal orientation (*OR* = 1.64, *P*<0.05) increased the likelihood of the student being regular drinking, compared to students with traditional orientations. Males (*OR* = 4.40, *P*<0.05) had a higher likelihood of regular drinking than females, graduate students (*OR* = 2.59, *P*<0.05) had a higher likelihood of regular drinking than undergraduates, students from urban areas (*OR* = 1.79, *P*<0.05) had a higher likelihood of regular drinking than those from towns/rural areas, and students attending key universities (*OR* = 0.48, *P*<0.05) had a lower likelihood of regular drinking than those attending general universities.

**Conclusions:**

Cultural orientation influences drinking behaviors. Traditional cultural orientation was associated with less drinking while western cultural orientation, marginal cultural orientation and bicultural orientation were associated with more drinking. The role of gender, hometown and university attendance is partially moderated through the influence of cultural orientation. The relationship between a traditional cultural orientation and alcohol drinking suggests that traditional Chinese cultural values should be examined for their role in possibly reducing alcohol-related risks through education and policy initiatives.

## Introduction

### Background

Reducing alcohol use, and high risk drinking patterns are important public health challenges, especially in countries experiencing rapid change. To develop effective programs to reduce alcohol related risks there is a need to better understand psychosocial and cultural influences on drinking behavior. In societies where alcohol is an integral part of social life and is largely unregulated by law it is especially important to understand the psychological and cultural aspects of drinking behavior [1].

In China, historical records indicate that alcohol has been used for thousands of years and is today an accepted part of daily life. Drinking patterns are deeply rooted in Chinese culture and daily life. Nevertheless, some patterns of alcohol use have detrimental effects on health. WHO [2] estimates that worldwide 4.5% of the global burden of disease and injury can be attributable to alcohol use. Serious social issues are also associated with alcohol use, including drunk-driving, violence, child neglect and abuse, and absenteeism in the workplace. In 1999 China ranked the second in the world for increases in per capita consumption of pure alcohol among people aged 15 years and older [3]. Between 1970–1972 and 2000–2005, the per capita consumption of alcohol, increased from 1.03 liters to 5.91 liters [2], [4]. Today China is the world’s largest producer of beer and liquor. Wine production in 2004 was up 15% from 2003 and the trend continues [5]. In 2011, WHO reported drinking trends to be stable. WHO estimated consumption of pure alcohol among those 15 years and older who drank to be 13.68 L of pure alcohol for males and for females 5.19 L of pure alcohol. In the same report they estimated that 9.6% of the drinkers were episodic heavy drinkers [2]. As in other countries, China faces public health and social consequences from excessive alcohol use, and understanding the psychological and sociological dimensions of this behavior will be useful to educational planners and policymakers.

### Cultural Orientation

When individuals living in their own society absorb new and different cultural values and attitudes as a result of exposure to media, education, cross-cultural relationships and foreign individuals and products their traditional cultural values are challenged. The degree to which an individual accepts or rejects the new or imported values relative to retaining their traditional indigenous value system reflects his or her cultural orientation [6], [7]. The concept of cultural orientation is distinct from acculturation. Cultural orientation describes a person’s changing cultural perspectives while they remain in their own society; acculturation is a measure of an individual’s acceptance of new cultural values as they move into a new society.

This study describes the cultural orientation of Chinese college students who are reconciling their traditional cultures with foreign cultures while remaining in their country of origin.

In the last three decades, following China’s opening to the west, there have been significant social, ideological, and cultural changes. At the same time alcohol drinking patterns have changed and traditional values have been increasingly challenged by outside influences especially western influences. Drinking behaviors often change along with changes in cultural practices and socio-cultural environment. Eide and Acuda et al [8]–[10] first examined the relationship between cultural orientation and alcohol use among secondary school students in Zimbabwe. They concluded that a western cultural orientation was associated with a higher probability for alcohol use and a local, traditional orientation was associated with a lower probability. Xue et al [6] and Shell et al [7] came to similar conclusions about the influence of cultural orientation on the alcohol use among Chinese college students.

### The Purpose of Our Study

We conducted a questionnaire survey among university students in China to examine the relationship between cultural orientation and drinking behavior. Also we took into account possible moderating factors like gender, hometown, grade and type of university attended. We believe that a better understanding of the psychosocial and cultural influences that affect drinking behavior can be useful to both researchers and public health practitioners working for drinking behavioral intervention and the betterment of related policies.

There are several reasons for choosing university students as our respondents. First, the college environment, at least in the west seems to support increased alcohol consumption [11]. Second, harmful drinking patterns, such as drinking to intoxication and binge drinking, seem to be on the rise among young adults[12]–[14], with serious, immediate and long term health risks [15]. Third, college youth in China are consistently exposed to western cultures through multiple channels, and thus have to cope with pressures from two or more cultures.

## Methods

### Ethical Consideration

This study was reviewed and approved by the ethical committee of School of Public Health, Tongji Medical College, Huazhong University of Science and Technology, Wuhan, China. The participation of this study was voluntary and confidentiality of the information assured. All participants signed an informed consent form prior to the investigation.

### Measures

#### Chinese Cultural Orientation Scale (CCOS)

Cultural orientation was measured by using the CCOS instrument, which contained 67 items representing ten subscales reflecting either Western or traditional cultural values. This instrument was developed over several years by the University of Nebraska-Lincoln and the Chinese Health Education Center [6], [7]. The CCOS uses a five-point Likert scale from strongly disagree (1) to strongly agree (5) to measure cultural orientation. Confirmatory factor analysis for the ten subscales showed an acceptable fit {Root mean square error of approximation (RMSEA) <0.06, standardized root mean square residual (SRMR) <0.08;see Hu&Bentler [16]}. Internal consistency tests showed that the reliabilities of most subscales were satisfactory, although Cronbach’s α values for consumerism and dating attitudes were low. Cronbach’s α values for each of the ten subscales, with sample statements from each of the subscale were as follows: 0.83 in gender discrimination (e.g. I think the male should play a leading role in my society), 0.76 in Chinese pride (e.g. I am proud of Chinese long history), 0.69 in appearance preference (e.g. I like having a cool hairstyle), 0.64 in interest in western culture (e.g. I appreciate the context of western culture that emphasizes on freedom and taking ease in life), 0.65 in filial piety (e.g. I would do what my parent wants me to do), 0.69 in the doctrine of mean (e.g. I won’t show off my strengths to avoid other’s repulsion), 0.56 in consumerism (e.g. I would rather buy something that makes me happy than deposit money in banks), 0.68 in respecting elders (e.g. I would sit down soon after elders have a seat), 0.69 in collectivism (e.g. I give priority to group interests other than individual ones), 0.53 in attitudes toward dating (e.g. I do not oppose sex before marriage as long as we love each other). For scoring, all items were coded so that a high score on traditional dimension means more traditional orientation and a high score on the western dimension means more western orientation.

Second-order factor analysis confirmed the bi-dimensional model of separate higher-order factors of westernization and traditionality found in previous work [7]. The bi-dimensional model assumes that cultural orientations are independent of each other (i.e., the degree to which one is oriented to traditional values is unrelated to the degree to which one is oriented to western values). Based on the bi-dimensional model, cultural orientations can fall into one of four orientations categories: traditional, western, bicultural and marginal [17]. Students reflecting a traditional cultural orientation hold strongly to traditional Chinese cultural values. Similarly students having a Western cultural orientation hold strongly to Western values. Students with a bicultural orientation hold strongly to both Chinese and Western values. Students with a marginal orientation do not hold strongly to either Western or traditional values. Second-order factor scores were computed by taking the mean of the subscale scores in each factor. We conducted a cluster analysis to confirm the four categories of cultural orientation and assess how many students fell into each category. The results showed (Table 1) that 486 (39.13%) students were western oriented, 405 (32.61%) were traditionally oriented, 214 (17.23%) were marginally oriented and 137 (11.03%) were biculturally oriented.

**Table 1:** The four categories in cultural orientations (N = 1242).

Categories	Number of students	Percentage (%)	Standard Mean Score	
			Traditional	Western
Marginally oriented	214	17.23	−1.39	−0.61
Western oriented	486	39.13	−0.20	0.59
Traditionally oriented	405	32.61	0.50	−0.82
Biculturally oriented	137	11.03	1.36	1.27
Total	1242	100.00	–	–

#### Drinking

Drinking status was assessed by measuring the drinking frequency within the last year and the last 30 days. Shell, Newman and Qian have used this measure to categorize drinkers and nondrinkers by drinking frequency in several studies [7], [18]–[19]. Participants were divided into three types of drinker: non-drinkers are those who reported never drinking or not drinking within the past year, occasional drinkers are those who drank in the last year but not in the past 30 days and those who drank 1 to 3 days in the past 30 days; and regular drinkers are those who drank 4 days or more in the past 30 days.

### Participants

Participants were a sample of 1279 students from six universities in Wuhan, China. Stratified cluster sampling was used to select the students. Strata were created based on gender. Student dormitory buildings were the secondary sampling units. In each university, one boy’s building was selected and one girl’s building was selected. From the six universities, twelve dormitory buildings were selected and each student of the buildings was asked to participate. Because of some no-response and some missing data, the total number of valid return was 1242 with a response rate of 97.11%. Of these, 634 (51.05%) were males and 608 (48.95%) were females. Mean age of the 1242 students was 21.22 years old (a range of 17–30 years old). Mean age was 21.57 years old (a range of 17–30 years old) for males and 20.85 years old (a range of 17–28 years old) for females; There were 365 (29.39%) freshmen, 91 (7.33%) sophomore, 402 (32.37%) junior, 220 (17.71%) senior and 164 (13.20%) graduate students; 902 (72.62%) students came from urban areas and 340 (27.38%) from towns or rural areas; 806 (64.90%) students came from key universities and 436 (35.10%) from general universities. In China, key universities are more prestigious, accepting students with higher entrance exam scores and receiving greater support from the central government.

### Procedures

This cross-sectional survey took place in 2011. Data collectors were university counselors or student leaders who had previous experience with conducting epidemiological surveys. All data collectors completed two days of training. Survey questions included information on demographic characteristics, alcohol drinking behavior and cultural orientation. Data collectors administered the questionnaire under the supervision of one of co-authors in this study. Emphasis was placed on answering the self-rated questionnaire independently, honestly and accurately. The questionnaire took approximately 30 minutes to complete.

### Analysis

Demographic differences and drinking behaviors in cultural orientations were examined using Chi-square test. Multinomial logistic regression analyses were performed to examine the relationship between cultural orientation and drinking behaviors, controlling for the socio-demographic characteristics of gender, grade, hometown and type of university. The significance set at *P*<0.05. We used SAS version 9.2 for all statistical analyses.

## Results

### Demographic Differences in Cultural Orientation

Chi-square measures of association were calculated to assess variations in cultural orientation among different socio-demographic groups. Table 2 presents cultural orientation by gender, grade, hometown and type of university attended. The distribution across the cultural orientation categories was found to be similar for graduates and undergraduates. In contrast, statistically significant differences in cultural orientation were observed for gender, hometown and type of university. Males were more likely to be western-oriented. Females were more likely to be traditional-oriented. Those who came from cities were more likely to be western-oriented, whereas students from town or rural areas were more likely to be traditional-oriented. Students in key universities were more likely to be western cultural oriented while students from general universities were more likely to be the traditionally cultural oriented.

**Table 2:** Demographic difference in cultural orientation (N = 1242).

Variables	Marginally oriented (n(%))	Western oriented (n(%))	Traditionally oriented (n(%))	Biculturally oriented (n(%))	*χ^2^*	*P*
**Gender**						
Male	126(19.87)	257(40.54)	174(27.44)	77(12.15)	17.9561	0.0004
Female	88(14.47)	229(37.66)	231(37.99)	60(9.87)		
**Grade** △						
Undergraduate	187(17.35)	417(38.68)	362(33.58)	112(10.39)	5.8889	0.1171
Graduate student	27(16.46)	69(42.07)	43(26.22)	25(15.24)		
**Hometown**						
Urban area	173(19.18)	363(40.24)	265(29.38)	101(11.20)	18.9325	0.0003
Town/rural area	41(12.06)	123(36.18)	140(41.18)	36(10.59)		
**University attendance**						
General university	59(13.53)	158(36.24)	161(36.93)	58(13.30)	13.7545	0.0033
Key university	155(19.23)	328(40.69)	244(30.27)	79(9.80)		

△ All the participants were divided into two groups (undergraduates and graduate students) in order to examine the drinking differences between undergraduates and graduate students, because we did not find the drinking differences in the four grades (freshman, sophomore, junior and senior) among undergraduates.

### Drinking Behaviors by Cultural Orientation

Table 3 presents the distribution of the four cultural orientation categories and the three drinking behaviors. Among all the students, there were 538 (43.32%) regular drinkers, 456 (36.71%) occasional drinkers and 248 (19.97%) nondrinkers. Chi-square measures showed differences in drinking behaviors by cultural orientation. Students with traditional orientation were more likely to be occasional drinkers or nondrinkers than their three counterparts. Students with marginal cultural orientation were more likely to be regular drinkers than their three counterparts. Bicultural-oriented students and western-oriented students were more likely to be regular drinkers than traditional-oriented students.

**Table 3:** Drinking behaviors by cultural orientation (N = 1242).

Cultural orientation	Regular drinkers (n(%))	Occasional drinkers (n(%))	Nondrinkers (n(%))	Total (n(%))	*χ^2^*	*P*
Marginally oriented	111(51.87)	67(31.31)	36(16.82)	214(17.23)	21.5470	0.0015
Western oriented	214(44.03)	172(35.39)	100(20.58)	486(39.13)		
Traditionally oriented	144(35.56)	167(41.23)	94(23.21)	405(32.61)		
Biculturally oriented	69(50.36)	50(36.50)	18(13.14)	137(11.03)		
Total	538(43.32)	456(36.71)	248(19.97)	1242(100.00)		

### Cultural Orientation and Drinking

Table 4 presents the odds ratios and 95% confidence intervals resulting from the multinomial logistic regressions. Comparisons of regular drinking and no drinking showed bicultural orientation increased the odds of regular drinking by 1.94 times, and marginal orientation increased the odds of regular drinking by 1.64 times, as compared to traditional orientation. The odds of males being regular drinkers were 4.40 times higher than females. Graduate students status increased the odds of regular drinking by 2.59 times relative to undergraduate students. The odds of being a regular drinker were 1.79 times higher for those who came from urban areas relative to those from town or rural areas. The odds of regular drinking were lowered by key university attendance, as compared to general university attendance. There were no significant differences between occasional drinking and non-drinking behaviors by cultural orientations, grades, home town and university attended; whereas, males had 1.74 times higher odds of occasional drinking versus no drinking than females.

**Table 4:** Odds ratios for drinking behaviors according to logistic regression (N = 1242).

Variables	Odds ratio	95% CI	*P*
Regular drinker versus non-drinker			
Marginal cultural orientation versus traditional cultural orientation	1.64	1.01,2.66	0.0443
Western cultural orientation versus traditional cultural orientation	1.18	0.81,1.71	0.3876
Bicultural cultural orientation versus traditional cultural orientation	1.94	1.06,3.54	0.0318
Gender(male versus female)	4.40	3.16,6.13	<0.0001
Graduate versus undergraduate	2.59	1.56,4.28	0.0002
Hometown(urban area versus town/rural area)	1.79	1.26,2.54	0.0012
Key university versus general university	0.48	0.33,0.71	0.0002
Occasional drinker versus non-drinker			
Marginal cultural orientation versus traditional cultural orientation	0.97	0.60,1.57	0.8903
Western cultural orientation versus traditional cultural orientation	0.92	0.64,1.31	0.6264
Bicultural cultural orientation versus traditional cultural orientation	1.44	0.79,2.63	0.2312
Gender(male versus female)	1.74	1.25,2.43	0.0010
Graduate versus undergraduate	0.96	0.56,1.65	0.8855
Hometown(urban area versus town/rural area)	1.29	0.92,1.81	0.1419
Key university versus general university	0.80	0.55,1.16	0.2405

## Discussion

Our findings indicate that cultural orientation is related to drinking behaviors among Chinese university students, similar to the findings of previous studies [7], [8], [20]. In addition, new evidence on the influence of cultural orientation on drinking emerged when we applied a bi-dimensional model with four cultural orientation categories. Traditional-oriented individuals were more likely to be occasional drinkers and nondrinkers than marginal-oriented, bicultural-oriented or western-oriented individuals. These findings suggest that a traditional cultural orientation is associated with less drinking and less risky drinking than other orientations. Consist with Herman-Stahl’s finding [20], when controlling for the socio-demographic factors, logistic regression indicated that being either a bicultural or marginal individual increased the odds of being a regular drinker relative to someone with a traditional cultural orientation. However, no significant differences were found between western and traditionally-oriented individuals after demographics were controlled.

Although the mechanism for how cultural orientation may affect patterns of alcohol use is not fully understood, there are some possible explanations. Traditionally oriented students possible drink less because they value traditional Chinese values, which evolved over time to support low risk drinking behavior. Values that did not support low risk drinking would threaten the survival of the society.

Counter intuitively and unlike some prior research [7], [8]–[10], students endorsing a Western orientation were not more likely to be either regular or occasional drinkers than students with a traditional orientation. Western cultural values come largely through media, advertising, and foreign visitors. This small window into Western culture tends to emphasize immediate gratification and pleasure, which would support increased alcohol. But, Western values also do not endorse some risky drinking behaviors that are part of traditional Chinese culture, like social and business toasting practices. Our findings also may be affected by using only a frequency measure for drinking. The difficulties with obtaining volume consumption measures in China have been well documented [18]–[19]. For college students in the West, the predominant risky drinking pattern is high levels of consumptions at infrequent intervals. It may be that the Western oriented students do not drink any more frequently than traditional oriented students but drink more when they drink. This requires further study.

It appears that the two mixed cultural orientations create the highest risk factors for problem drinking. In both orientations, individuals do not have a clear identity. Bicultural individuals who endorse values from both the receiving and traditional cultural contexts may find themselves without a clear value system and able to vary their position depending on the circumstances [21]. Marginal individuals who reject the foreign culture and do not value their traditional cultural values lack any firm value system for guiding behavior [2]. It has been suggested that due to stress created by the conflicts associated with being marginal or bicultural oriented individuals might be motivated to escape such stress or conflict by consuming more alcohol [22]–[23].

Our findings confirmed gender differences in alcohol use, that is, males tend to be higher frequent drinkers, similar to the findings of Shell and colleagues [7]. Much of the variation in gender differences in drinking can also be linked to culture [1]. Our investigation found that females were more likely to be traditional-oriented than males. This suggests that gender differences may be mediated through differences in cultural orientation [24].

Graduate students were more likely to be regular drinkers than undergraduates. A scant amount of literature has shown the specific differences between the two populations. Our findings confirm the findings of Wang and Zhang’s study among university students in Beijing that graduate students were prone to be to drink more [25]. Our findings indicate that these differences do not appear to be due to different cultural orientations in the two groups. Further study is needed to examine the origin of these differences.

Students who came from an urban area tended to be higher frequent drinkers than those from town/rural area. Zhou’s study in Hunan Province in China showed that the drinking rates were higher in the urban area (45.9%) than in the rural area (39.6%) [26]. It is difficult to specifically assess the reasons for the differences in drinking behaviors between urban and rural population, however, with our findings that students from town/rural area were more likely to be traditional oriented, this is most likely due to a combination of the different cultural background and other complicated factors, such as levels of economic development, working environment, educational background and etc.

Key university attendance was associated with less drinking than general university attendance. Key universities refer to universities recognized as prestigious and which received a high level of support from the central government with different campus culture and model of teaching from general universities. Our findings suggest the type of university attended was associated with cultural orientation. Students in key university were more likely to be western-oriented. Despite this they drank less, possibly the result of the heavy time requirements for academic success that leave little time for recreational activities that might include alcohol use. It may be the case that key students, like students in Western colleges, drink fewer times but drink more each time they drink. This possibility clearly needs further study.

In summary, culture orientation had an important impact on drinking behaviors. The role of gender, hometown and university type on drinking behaviors is partially mediated through the influences of culture orientation. The significance of our findings could be considered as the basis for alcohol policy and program development. These factors that differentially contribute to alcohol use deserve careful and detailed attention in efforts to reduce alcohol drinking. Reinforcing traditional beliefs and values may be beneficial for less drinking. In the meantime, significant changes in the formal policy environment at the university level seem to be indispensable. Specific strategies of preventive intervention would meet specific population, considering the role of gender, hometown, campus environment and graduate students/undergraduates.

Several limitations of this study should be mentioned. First, it should be noted that this study only took place in a single city in China. Whether the findings would generalize to other parts of China is unknown. Second, the study is cross-sectional. Data on drinking patterns over time and cultural orientation changes over time may provide new insights into their relationship. Finally, the continued development of the CCOS is also needed to enhance reliability and validity. At the same time a more detailed analysis of the relationships of drinking and other variables could help in understanding problematic drinking pattern [27]. Given these limitations, our findings need to be replicated and refined in future studies. More researches are encouraged to examine the deep relationship between specific aspects of cultural orientation and drinking patterns, determine what protective factors are provided by culture, and investigate marginal and bicultural individuals and the possible role stress plays in drinking behavior.
